# Enhanced Positioning Strategies to Reduce Pneumothorax in CT-Guided Lung Biopsies

**DOI:** 10.3390/diagnostics14232639

**Published:** 2024-11-23

**Authors:** Michael P. Brönnimann, Leonie Manser, Martin H. Maurer, Bernhard Gebauer, Timo A. Auer, Dirk Schnapauff, Federico Collettini, Thanh-Long Nguyen, Alois Komarek, Miltiadis E. Krokidis, Johannes T. Heverhagen

**Affiliations:** 1 Department of Diagnostic, Interventional and Pediatric Radiology, Inselspital, Bern University Hospital, University of Bern, Freiburgstrasse 10, 3010 Bern, Switzerland; leonie.manser@insel.ch (L.M.); alois.komarek@insel.ch (A.K.); miltiadis.krokidis@insel.ch (M.E.K.); johannes.heverhagen@insel.ch (J.T.H.); 2 Department of Radiology, Charité—Universitätsmedizin Berlin, Augustenburger Platz 1, 13353 Berlin, Germany; bernhard.gebauer@charite.de (B.G.); timo-alexander.auer@charite.de (T.A.A.); dirk.schnapauff@charite.de (D.S.); federico.collettini@charite.de (F.C.); 3 Department of Diagnostic and Interventional Radiology, Oldenburg University Hospital, Carl von Ossietzky University of Oldenburg, 26129 Oldenburg, Germany; martin.maurer@uni-oldenburg.de; 4 Clinician Scientist Program, Berlin Institute of Health, Charité—Universitätsmedizin Berlin, 13353 Berlin, Germany; 5 Department of Thoracic Surgery, Inselspital, Bern University Hospital, University of Bern, 3010 Bern, Switzerland; thanh-long.nguyen@insel.ch; 6 1st Department of Radiology, School of Medicine, National and Kapodistrian University of Athens, Areteion Hospital, 15772 Athens, Greece

**Keywords:** pneumothorax, biopsy, image-guided biopsy, risk factors, tomography, lung, risk factors

## Abstract

Background/Objectives: This study aimed to investigate pneumothorax risk, focusing on the gravitational effect of pleural pressure caused by specific patient positioning. Methods: We retrospectively analyzed 144 percutaneous CT-guided lung biopsies performed between January 2019 and December 2023. Patients were grouped into those with or without pneumothorax. Variations in patient positioning (prone, supine, lateral, lesion in decubitus biopsy-side-down [LD BSD] and the dependent area [L DA M], and access route beginning in the dependent area [AR LD M]) were compared using the chi-square, Fisher’s exact, and Mann–Whitney U tests. Performance metrics were evaluated. Univariate and binomial logistic regression models assessed the influence of these factors and other patient-related and interventional parameters on pneumothorax occurrence. Results: Three positional variants (AR DA M, L DA M, and L LD BSD; *p* < 0.001), general emphysema (*p* = 0.009), emphysema in the access route (*p* = 0.025), greater needle size (18G vs. 20G; *p* < 0.001), and the use of a side-cut instead of a full-core system (*p* = 0.002) were significantly linked to lower peri-interventional pneumothorax incidence. Even after adjusting for various factors, AR DA M and general emphysema remained independently associated with a reduced pneumothorax risk (OR 0.168, *p* < 0.001; OR 2.72, *p* = 0.034). Assessing the dependent zones showed superior performance regardless of the patient’s position, with the best performance demonstrated for AR DA M (AUC 0.705; sensitivity 60%, specificity 81.8%). Conclusions: Focusing on the dependent zones of each lung and adjusting the access route accordingly can significantly reduce the risk of pneumothorax compared to conventional positioning techniques.

## 1. Introduction

The most frequent complication of CT-guided lung biopsies is pneumothorax, occurring when subatmospheric pleural pressure, created by the stiff chest wall and the lung’s elastic recoil force, is neutralized by infiltrating air [1,2,3]. It is reported that this frequency ranges from 8% to 69% [1,2,3,4,5,6,7,8,9,10,11,12,13,14,15]. Most pneumothoraces caused by this procedure are small, asymptomatic, and resolve spontaneously. However, larger pneumothoraces require the placement of a drainage catheter (5–15%), additional imaging, and hospital admission. These complications not only jeopardize patient safety but also increase healthcare costs significantly, including expenses for catheter insertion, follow-up imaging (e.g., chest X-rays or CT scans), extended hospital stays, and potentially longer recovery periods. The overall cost increase can range from 300% to 400%, imposing a substantial economic burden on the healthcare system [8,10,13,16,17,18,19,20,21,22,23,24].

Patient positioning during CT-guided lung biopsy has been extensively investigated, yielding conflicting but promising results. Some studies indicate that performing the biopsy with the patient in the biopsy-side-down position does not reduce the risk of pneumothorax [20,25], while the PEARL approach [26] suggests the opposite. Other research has shown a reduction in pneumothorax risk when the patient is moved to the dependent, biopsy-side-down position after the biopsy [6,7,8,27]. However, Leger et al. found no evidence to support this [20]. Preventive positioning prior to biopsy has only been evaluated by a few authors [25,28,29].

These discrepancies may be attributed to the pleural cavity’s physiological characteristics, which exhibit stronger negative pressure in the higher, non-dependent lung regions [27]. Ipsilateral patient positioning may be relevant by ensuring that the needle path passes entirely through dependent lung regions [28].

**Figure 1:**
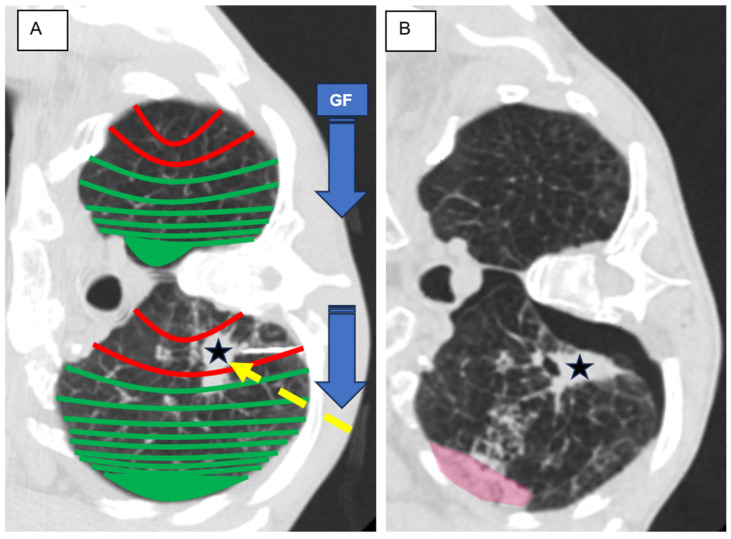
Graphical abstract—schematic illustration of the influence of gravitational force on the lung tissue. (**A**) The patient was positioned in the lateral decubitus with biopsy-side-down to prevent pneumothorax. Coaxial needle with tip near the target lesion (asterisk). Access route and lesion location in the non-dependent area. In contrast, it is possibly the safer access route (dotted yellow arrow). (**B**) After a full-core lung biopsy, a pneumothorax and higher-grade pulmonary hemorrhage (red area) resulted. GF = gravitational force.

This study aimed to investigate the risk of pneumothorax, focusing on the gravitational effect of pleural pressure caused by specific patient positioning (Figure 1). Specifically, it sought to determine the ideal patient position to prevent pneumothorax through direct comparison and performance measurement.

## 2. Materials and Methods

**Figure 2:**
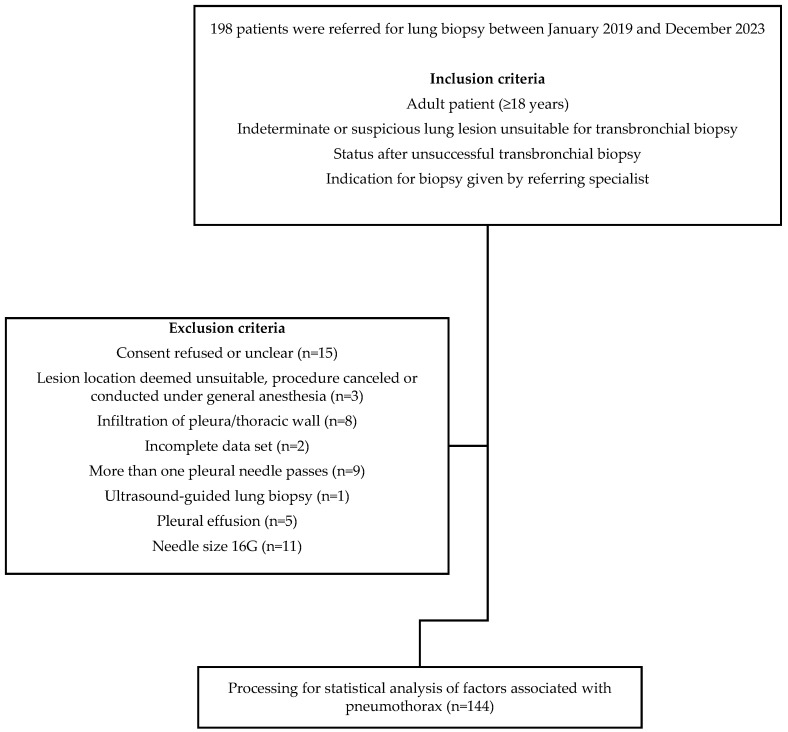
Flowchart showing the study population.

This retrospective analysis included 198 percutaneous CT-guided lung biopsies performed at our university hospital between January 2019 and December 2023. Biopsies using 16-gauge needles were excluded, as needles larger than 18-gauge are considered a significant risk factor for the occurrence of pneumothorax [30]. Biopsies performed under general anesthesia were also excluded due to the well-documented higher risk of pneumothorax. Other specific exclusion criteria were consecutively applied to avoid compromising the results due to the predominant abnormal physiology in the pleural cavity (Figure 2).

### 2.1. Baseline Evaluation and Biopsy Technique

All patients underwent a clinical examination prior to the procedure, which included a detailed medical history and standard blood tests. An INR value below 1.5, a quick value above 60%, a Hb value above 80 g/L, and a platelet count greater than 50 × 10^9^/L were required for the intervention. Four board-certified, independent, interventional radiologists, two with seven years of experience each and two with more than 15 years of experience, performed all lung biopsies in equal numbers. Each interventionalist could decide how to position the patient for the biopsy. The procedures were performed with CT guidance using a Toshiba Asteion 4SL and a 17 or 19-gauge coaxial needle, along with an 18- or 20-gauge semiautomated biopsy system (SemiCut side-cutting; Medical Devices Lease S.A., Zug, Switzerland, or CorVocetTM full-core; Merit Medical Systems, South Jordan, UT, USA). A non-contrast chest CT was obtained and reconstructed at 1 mm increments to plan the biopsy, with the needle path planned according to the current gold standard. The skin, subcutaneous tissues, and parietal pleura were locally anesthetized with 1% lidocaine (maximum 20 mL). Breathing commands were not given, as studies have shown that hyperventilation phases often occur as compensation after the initial commands, prolonging the procedure [31]. After the tissue sampling was completed, the needle was quickly removed without using a sealing agent (blood patch). A control CT scan was performed, and if no complications occurred, the patient was transferred to a bed in the supine position. There was no transfer to the biopsy site. A drainage system (Safe-T-Centesis TM 6 or 8F) was used if a progressive pneumothorax was detected on the control CT scan immediately after needle withdrawal and five minutes later. All patients were monitored (routine vital signs) for four hours after the procedure on the ward. If the patients showed no complications after the monitoring, they were discharged home. If the control CT scan showed a non-progressive pneumothorax, a chest X-ray was performed six hours later for further assessment. If the pneumothorax exceeded 2 cm apical, the patient was admitted as an inpatient for one night.

### 2.2. Differentiation Between Biopsy in Dependent and Non-Dependent Areas of the Lung

**Figure 3:**
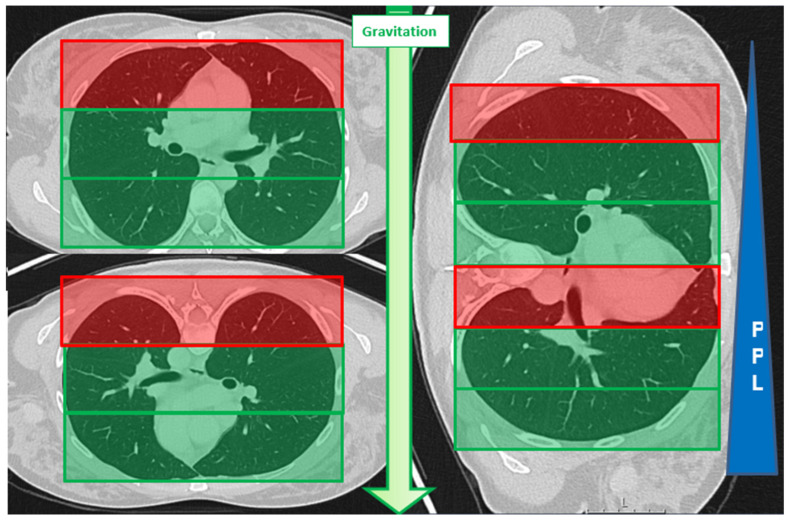
Schematic illustration of the zoning used for this study according to position-dependent gravitational effect on pleural pressure (PPL). From non-dependent in the upper to dependent lung regions lower down, the PPL increases. However, in a healthy, non-intubated lung, PPL always remains negative [32]. The weight of the lung influences the PPL, and an additional force acts into the periphery. Consecutively and for simplification, we determined only the zone “RED” as non-dependent. For zoning, we applied the rule of thirds.

We applied our previously tested, simplified zoning model by dividing the axial planning images into three-thirds and using non-anatomical landmarks for orientation [28]. The red zone, defined as the non-dependent lung area, was the third in which gravitational force was strongest. The remaining two-thirds were evaluated as dependent lung areas (Figure 3). According to the schematic representation of the gravitational effect on pleural pressure by Stenqvist et al. [32], pleural pressure is considered positive from the middle third downward due to progressive lung collapse.

**Figure 4:**
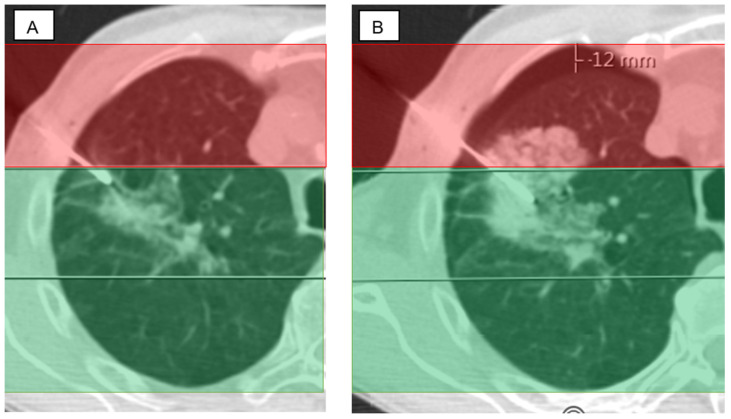
Discrepancy between access route and lesion location according to our zoning model. (**A**) The patient is in a supine position with a target lesion in the dependent area. The access route starts in a non-dependent area. (**B**) Already, during the lung biopsy, a pneumothorax occurred with a width of 12 mm.

In addition, the position of the target lesion and the entry point of the coaxial needle into the lung were assessed (Figure 4).

### 2.3. Procedures and Patient Groups

All procedures were reviewed by a board-certified interventional radiologist with eight years of experience and a radiology resident with three years of experience, both of whom were blinded to the patient’s medical history and did not perform any interventions. All interventional images were analyzed using Sectra Workstation software (Model IDS7, Version 24.2, Patch 4/2022; Sectra AB, Linköping, Sweden). The patients were categorized based on the occurrence of pneumothorax. We recorded various factors, including patient demographics, positioning during the biopsy (supine, prone, or lateral decubitus with biopsy-side down, abbreviated as L LD BSD), and lesion location. The access route and lesion location were classified according to our zone model, where “L DA M” indicates the lesion is in a dependent area, and “AR DA M” signifies the access route is in a dependent area. Additional details that were recorded were lesion size, distance from the skin and pleura to the lesion along the needle pathway, biopsy angle, needle size and type, number of samples, procedure duration (measured as the time in minutes from the first to the last image taken), and the name of the interventionalist performing the biopsy. Additionally, it was visually assessed whether emphysema was present in general or along the access route in the lung window. These interventional-specific parameters were evaluated based on the interventional images and recorded from the intervention report. Histological results from the target lesion and the patient’s post-interventional history were collected retrospectively from electronic medical records.

### 2.4. Statistical Analysis

Statistical analyses were conducted using commercially available software (IBM SPSS Statistics for Windows, version 28; IBM, Armonk, NY, USA). Categorical variables were analyzed using the chi-square and Fisher’s exact tests, while continuous variables were assessed using the Mann–Whitney U test. The Kolmogorov–Smirnov test determined normal distribution. Spearman’s correlation was used for continuous variables, and contingency correlation was used for the categorical values to identify high correlations. The phi coefficient evaluated the categorical variables, and Pearson’s correlation coefficient determined the effect size for continuous variables. In cases of high correlation, only variables with the highest effect size were included in the logistic regression model. The Kruskal–Wallis test was used to determine a significant time difference between the different positioning groups. The optimal measurement method aimed for high sensitivity and AUC with minimal false negatives. All tests were two-sided with a significance level of *p* < 0.05. To prevent overfitting, the logistic regression model adhered to the rule of ten [33], with a minimum group size of n ≥ 25 for the categorical predictors. The logistic regression model assessed potential risk factors for pneumothorax [34]. Variables with a *p*-value of <0.1 in the univariate analysis were included in the multivariate regression model.

## 3. Results

### 3.1. Study Population

**Figure 5:**
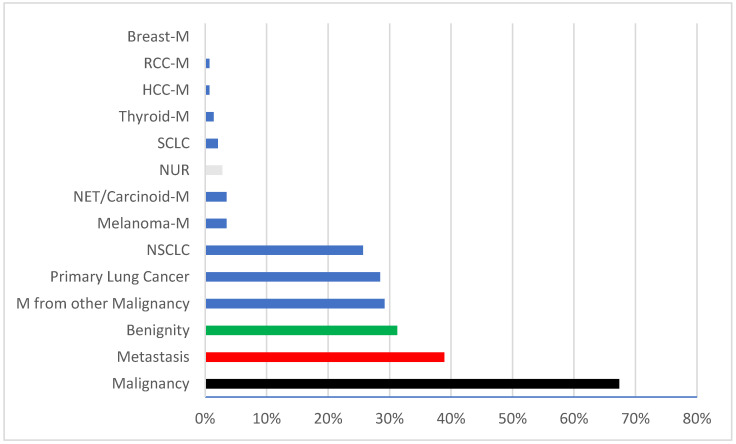
Histological findings of the lung biopsies. M = metastasis; RCC = renal cell cancer; HCC = hepatocellular carcinoma; SCLC = small cell lung cancer; NUR = no usable result; NET = neuroendocrine tumor; NSCLC = non-small cell lung cancer.

Our final study population included 144 patients enrolled after the application of our inclusion/exclusion criteria, were, on average, 65.91 ± 13.15 years old, the youngest at 18 and the oldest at 89 years, and 40% of patients were women. A total of 67% of the biopsies were malignant, with metastases (39%) as the most frequent representative. Only one-third of the cases resulted in benign findings (31%) (Figure 5). After histologic confirmation of malignancy, 60% of the patients received anticancer therapy; of these, 83.3% underwent surgical resection, while the remaining patients received adjuvant systemic therapy.

Age, sex, lesion size, patient position, lesion location, number of samples, procedure time, biopsy angle, skin-to-lesion, pleura-to-lesion distance, and pathological findings did not differ significantly between patients (Table 1). The analyzed variables were not normally distributed in the groups.

### 3.2. Pneumothorax After CT-Guided Lung Biopsy

**Figure 6:**
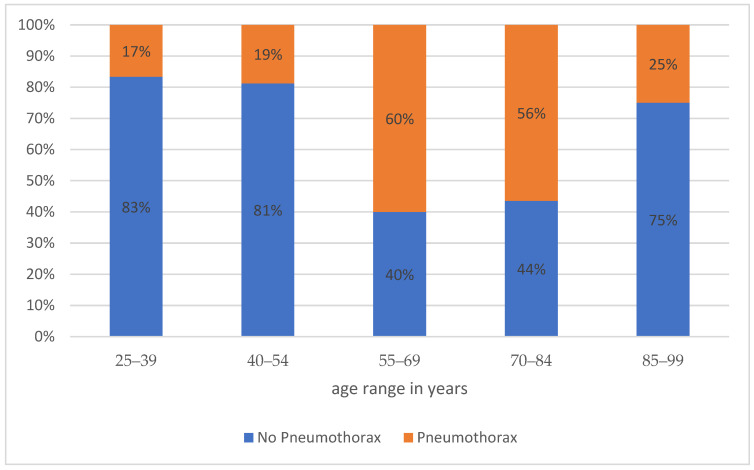
Frequency distribution of pneumothorax occurrence by age group.

The pneumothorax rate was 51.3%; in 7.6% (11/144), drainage had to be inserted. Of these 11 cases, 8 showed visual evidence of emphysema both generally and along the access route. A success rate of 94.4% resulted. A total of 60% of pneumothoraces occurred in the age group between 55–69 years and 56% in the age group between 70–84 years (Figure 6). In all other age groups, the proportion of biopsies without complications predominated.

The univariate analysis revealed that pneumothorax occurred significantly more frequently when general emphysema (*p* = 0.009) or emphysema in the access route was present (*p* < 0.01) and when a larger needle (18G) (*p* < 0.01) and a full-core biopsy system (*p* = 0.002) were used. The lowest incidence of pneumothorax occurred when lesions were biopsied in dependent areas (19/74; 26%, *p* < 0.01), when the access route started in a dependent area (14/74; 19%, *p* < 0.01), or when the lesion was in the biopsy-side-down position (patient in lateral decubitus) (2/74; 2%, *p* < 0.01). The phi correlation indicated that the strongest association with a post-biopsy pneumothorax was when the access route started in a dependent area (Table 1). We did not find a significantly reduced frequency of drainage insertions by different positioning variants (supine: *p* = 0.749; prone: *p* = 1.000; L DA: *p* = 0.755; AR DA: *p* = 0.529; L LD BSD: *p* = 0.228).

### 3.3. Association Lesion Characteristics and Technical Parameters with the Occurrence of Pneumothorax

The contingency correlation analysis revealed that the analyzed parameters of the positional variants and the visual assessment of the emphysema were highly correlated (*p* < 0.01). Consequently, it was decided to include only the access route in the dependent area and general emphysema in the logistic regression model. Binomial logistic regression analysis showed that the access route starting in the dependent area (OR 0.168, 95% CI 0.068–0.415, *p* < 0.001) and with present general emphysema (OR 2.72, 95% CI 1.076–6.986, *p* < 0.001) were independently associated with a higher incidence of pneumothorax (Table 2). A moderate model fit was obtained with an R^2^ = 0.332, *p* < 0.01; Cohen’s f2 was 0.49, corresponding to a strong effect [35]. For completeness, we performed the binomial logistic regression separately for the other highly correlated variables, but each time with a significantly worse model fit and effect size.

### 3.4. Performance Metrics of Positional Variants to Prevent a Pneumothorax

**Figure 7:**
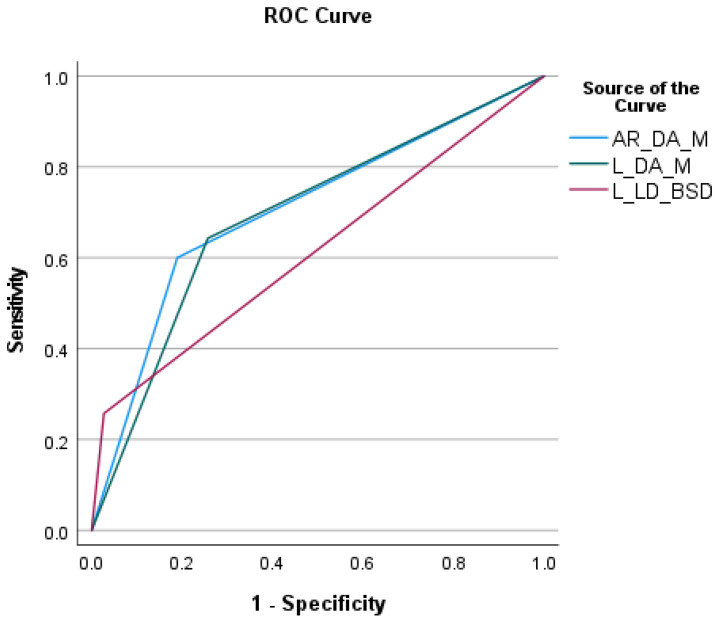
Receiver operating curve (ROC) of the positional variants to prevent pneumothorax. AR DA M = access route in dependent area according to model; L DA M = lesion in dependent area according to model; L LD BSD = lesion location in lateral decubitus biopsy-side-down position.

The investigated positional variants to prevent a pneumothorax showed that it provided the best results when the access route begins in the dependent area (AUC 0.705, sensitivity 60%, and specificity 81.10%). In particular, the additional subdivision into dependent areas increased the sensitivity by almost 40% (from 25.7 to 64.3% resp. 60%) and reduced false-negative predictions from 52 to 25 (L LD BSD vs. lesion DA) resp., and from 52 to 28 (L LD BSD vs. AR DA) (Figure 7 and Table 3). The different patient positions did not lead to any significant procedure time difference (supine: 27 ± 8.041 min; prone = 25.05 ± 7.174 min; LD BSD = 25.54 ± 8.558 min, *p* = 0.140).

## 4. Discussion

This study showed, for the first time, that starting the access route in the dependent area resulted in a 5.95-fold risk reduction for the occurrence of pneumothorax (*p* < 0.001). Additionally, an assessment of the dependent zones proved superior, regardless of the patient’s position, with the best performance for the former (AUC 0.705; sensitivity 60%, specificity 81.80%). Visual detection of generalized emphysema was independently associated with a 2.72-fold increased risk for post-interventional pneumothorax (*p* = 0.034). This finding is crucial, as targeted patient positioning can avoid non-dependent areas.

The findings of this study corroborate and extend the findings of other research groups. For instance, a canine study showed that positioning dogs in the biopsy-side-down decubitus position halted pneumothorax progression [27]. Cassel et al. [6] demonstrated a reduction in pneumothorax rates by immediately positioning patients (n = 80) on the puncture side post-procedure. Drumm et al. [25] and subsequently Najafi et al. [26], through prospective data, showed that positioning patients in the biopsy-down (ipsilateral decubitus) position as part of the PEARL protocol decreased pneumothorax incidence. Zidulka et al. [27] suggested that positioning patients in the dependent position could reduce pressure differences between the alveoli and the pleura and decrease alveolar size, thereby potentially reducing the incidence of pneumothorax. This hypothesis is grounded in the physiological dynamics of the pleural cavity: gravitational forces in dependent lung regions assist in maintaining lung expansion, which may mitigate the development of subatmospheric pressure gradients that contribute to pneumothorax formation during needle insertion [20]. Our results further indicate that the critical alteration in force effect occurs at lung entry. Thus, it appears that the access route’s trajectory plays a decisive role rather than the lesion’s final position (L DA M, *p* ≤ 0.001, φ −0.388, AUC 0.693 vs. AR DA M, *p* ≤ 0.001, φ −0.421, AUC 0.705).

The absence of influence of the positional variations on procedure time and drain placement aligns with the existing literature. Drumm et al. [25] found no significant difference in the chest tube placement rates between positioning groups. O’Neill et al. [22] concluded that the relatively low occurrence in the prone or supine positions might suggest that these patients were promptly repositioned to the biopsy-side-down position after the procedure, a practice known to reduce the need for tube placement. However, our results instead indicate that the development of a severe pneumothorax is influenced by the physiological correlations mentioned in the previous section rather than by the rollover maneuvers itself. A direct comparison in a future study is desirable. Drumm et al. already believed that performing procedures with the biopsy side down does not require additional time, a finding that our study has confirmed [25].

We found a significantly higher incidence of a pneumothorax when in the access route (*p* = 0.025) or in general when emphysema (*p* = 0.009) was present, and an independent association of the latter with an increased risk for pneumothorax (*p* = 0.034). We support the findings of Lee et al. [36] (visual method) and Zhang et al. [37] (quantitative method), both of whom demonstrated that perilesional emphysema serves as an independent predictor for pneumothorax. We are also in line with the findings for general emphysema, as Theilig et al. [38] reported, that quantitatively determined pulmonary emphysema was a positive predictor of pneumothorax risk. However, the literature shows inconsistent findings in the subjective assessment of this circumstance (e.g., Lee et al. [36], Anderson et al. [39], Yeow et al. [40], and Asai et al. [5]). We believe that further research is needed to directly compare the visual and quantitative measurement methods for emphysema detection to understand better the impact of general or perilesional parenchymal destruction on pneumothorax risk.

We observed a trend towards a lower incidence of pneumothorax in patients aged 70 years or older, aligning with the findings by Wiener et al. [41] and Vatrella et al. [42]. This may be explained by the decreased elastic recoil in the lung parenchyma of older patients (e.g., [32,42]). On the other hand, the lower internal tension could be a contributing factor [43]. The higher pneumothorax rate (51.3%) observed in our study is largely due to our stringent criteria for pneumothorax detection. We classified any degree of air accumulation in the pleural cavity on post-biopsy CT as pneumothorax, irrespective of its extent. This approach contrasts with other studies where the detection methods may be less sensitive or when only more advanced pneumothoraces are reported. For instance, some authors rely on posteroanterior chest radiographs, which may under-detect smaller or asymptomatic pneumothoraces. Other studies consider only pneumothoraces that result in a complete circumferential detachment of the visceral pleura, excluding minor air accumulations from their reported rates. By using post-biopsy CT as our primary diagnostic tool, we likely identified smaller or subclinical pneumothoraces that might not be visible on chest radiographs or were disregarded in other studies due to their limited clinical significance. This difference in detection methodology underscores the variability in reported pneumothorax rates across studies and highlights the impact of imaging modality and classification criteria on pneumothorax incidence in lung biopsy research (e.g., [42]).

Limitations include the retrospective nature and single-center scope of this study, lack of external validation, and a simplified model of pleural pressure’s gravitational effect. A future prospective study could further quantify this effect.

## 5. Conclusions

Focusing on the dependent zones of each lung and adapting the access route can significantly reduce a pneumothorax risk compared to conventional patient positioning techniques. This method highlights the importance of customized procedural planning in interventional radiology. The clinical practice in our center has evolved based on our findings, leading to a protocol change where patients are now positioned to ensure that the biopsy access route traverses dependent lung regions whenever possible. These insights into optimal access routes and emphysema management have significantly enhanced procedural safety and improved patient outcomes.

## Figures and Tables

**Table 1:** Patient demographics and lesion characteristics. Unless stated otherwise, data are the number of biopsies. ± standard deviations. X2 (R X 2), Fisher’s exact test and the Mann–Whitney U test were used to calculate the statistical difference between groups of categorical, dichotomous, and continuous variables, respectively. Data are mean ± standard deviation. * Statistically signifianct defined *p* < 0.05. Y = year; mm = millimeter; SL = skin-to-lesion; PL= pleural to lesion; ML/L = middle lobe or lingula; UL = upper lobe; LL = lower lobe; UL = upper lobe; LL = lower lobe; L DA M = lesion in dependent area according to our model; LD BSD = lateral decubitus biopsy-side-down; AR DA M = access route begins in dependent area according to our model; G = gauge; min = minutes; NUR = no usable result; φ = phi coefficient; CC = contingency coefficient.

Survey of Lung Biopsies									
Parameter	All (n = 144)		No Pneumothorax (n = 70)		Pneumothorax (n = 74)		*p*-Value	φ	CC
Female	58	40%	33	47%	25	34%	0.127		
Age (y)	65.9	±13.15	64.19	±14.87	67.54	±11.16	0.217		
Lesion Size (mm)	27.2	±20.06	28.79	±19.49	25.62	±20.60	0.148		
Patient Position							0.051		<0.01
Supine	55	38%	27	39%	28	38%			
Lateral	46	32%	28	40%	18	24%			
Prone	42	29%	15	21%	27	36%			
L DA M	64	44%	45	64%	19	26%	<0.001 *	−0.388	<0.01
LD BSD	20	14%	18	26%	2	3%	<0.001 *	−0.333	<0.01
AR DA M	56	39%	42	60%	14	19%	<0.001 *	−0.421	
General Emphysema	53	37%	18	26%	35	47%	0.009 *	0.224	
Emphysema LAR	40	28%	13	19%	27	36%	0.025 *	0.200	<0.01
Lesion Location							0.924		
UL	65	45%	31	44%	34	46%			
LL	72	50%	35	50%	37	50%			
ML/L	7	5%	4	6%	3	4%			
Needle Size							<0.001 *		
18 G	101	70%	40	57%	61	82%			
20 G	43	30%	30	43%	13	18%			
Biopsy System							0.002		
side-cut	77	53%	50	71%	25	34%			
full-core	43	30%	15	21%	28	38%			
Number of Samples (n)							0.865		
1 and 2	27	19%	14	20%	13	18%			
3	64	44%	32	46%	32	43%			
4	34	24%	15	21%	19	26%			
5	17	12%	9	13%	8	11%			
Procedure Time (min)	26.3	±8.00	24.89	±7.49	27.59	±8.30	0.055		
Biopsy Angle (degree)	63.8	±18.33	65.2	±18.26	62.43	±18.43	0.369		
Distance SL (mm)	59.7	±20.54	62.5	±22.05	56.96	±18.76	0.159		
Distance PL (mm)	14.9	±14.96	14.6	±13.90	15.14	±15.99	0.774		
Pathological Findings							0.822		
Primary Lung Cancer	41	36%	20	32%	21	40%			
Metastasis	54	47%	28	45%	26	49%			
Benignity	45	39%	21	34%	24	45%			
NUR	4	3%	1	2%	3	6%			

**Table 2:** Binomial logistic regression predicting the likelihood of pneumothorax. The total number of cases in the cohort for the multivariable analysis was n = 144. B = regression coefficient; S.E. = standard error; df = degree of freedom; CI = confidence interval; AR DA M = access route in dependent area according to model; G = gauge; * statistically signifianct defined *p* < 0.05.

Variable	B	S.E.	Wald Test	df	*p*-Value	Odds Ratio	95% CI	
							−	+
Procedure Time (min)	0.02	0.029	0.705	1	0.401	1.024	0.968	1.084
AR DA M	−1.78	0.461	14.955	1	<0.001 *	0.168	0.068	0.415
General Emphysema	1.01	0.477	4.47	1	0.034	2.72	1.076	6.986
Biopsy System	−0.57	0.511	1.258	1	0.262	0.564	0.207	1.535
Needle Size	0.54	0.528	1.059	1	0.303	1.722	0.612	4.85

**Table 3:** Performance metrics of different positional variants to prevent pneumothorax. AR DA M = access route in dependent area according to our model; L DA M = lesion in dependent area according to our model; L LD BSD = lesion location in lateral decubitus biopsy-side-down position; AUC = area under the curve.

Positional Variant	AUC	Sensitivity	Specificity	False-Negative	False-Positive
AR DA M	0.705	60%	81.10%	40% (28/70)	18.9% (14/74)
L DA M	0.693	64.30%	74.30%	35.7% (25/70)	25.7% (19/74)
L LD BSD	0.615	25.70%	97.30%	74.3% (52/70)	2.7% (2/74)

## Data Availability

The dataset is available upon request from the authors.
